# Coronary risk factor profiles according to different age categories in premature coronary artery disease patients who have undergone percutaneous coronary intervention

**DOI:** 10.1038/s41598-024-53539-6

**Published:** 2024-07-03

**Authors:** Sukma Azureen Nazli, Azhari Rosman, Noor Alicezah Mohd Kasim, Alyaa Al-Khateeb, Ahmad Zia Ul-Saufie, Ahmad Bakhtiar Md Radzi, Khairul Shafiq Ibrahim, Sazzli Shahlan Kasim, Hapizah Nawawi

**Affiliations:** 1 Institute of Pathology, Laboratory and Forensic Medicine (I-PPerForM), Universiti Teknologi MARA, Selangor, Malaysia; 2 Faculty of Medicine, Universiti Teknologi MARA, Selangor, Malaysia; 3 Cardiac Vascular and Lung Research Institute (CaVaLRI), Universiti Teknologi MARA, Selangor, Malaysia; 4 Faculty of Computer and Mathematical Sciences, Universiti Teknologi MARA, Selangor, Malaysia; 5 Institut Jantung Negara (IJN), Kuala Lumpur, Malaysia

**Keywords:** Cardiology, Health care, Risk factors

## Abstract

Many studies have investigated the coronary risk factors (CRFs) among premature coronary artery disease (PCAD) patients. However, reports on the proportion and CRFs of PCAD according to different age cut-offs for PCAD is globally under-reported. This study aimed to determine the proportion of PCAD patients and analyse the significant CRFs according to different age cut-offs among percutaneous coronary intervention (PCI)-treated patients. Patients who underwent PCI between 2007 and 2018 in two cardiology centres were included (n = 29,241) and were grouped into four age cut-off groups that defines PCAD: (A) Males/females: < 45, (B) Males: < 50; Females: < 55, (C) Males: < 55; Females: < 60 and (D) Males: < 55; Females: < 65 years old. The average proportion of PCAD was 28%; 9.2% for group (A), 21.5% for group (B), 38.6% and 41.9% for group (C) and (D), respectively. The top three CRFs of PCAD were LDL-c level, TC level and hypertension (HTN). Malay ethnicity, smoking, obesity, family history of PCAD, TC level and history of MI were the independent predictors of PCAD across all age groups. The proportion of PCAD in Malaysia is higher compared to other studies. The most significant risk factors of PCAD are LDL-c, TC levels and HTN. Early prevention, detection and management of the modifiable risk factors are highly warranted to prevent PCAD.

## Introduction

Cardiovascular disease (CVD) is known as the leading cause of death globally. Despite the improvements in the healthcare system, coronary artery disease (CAD) remains among the top causes of disease, disability and death worldwide leading to a high consumption of health resources. Even though it was reported that the trend in CVD incidence and mortality rates is declining and the survival rate of CVD patients is stable^1^, the concern is that the age of onset of CAD in Malaysia is younger compared to other developed countries^2,3^. Various Southeast Asian registries have reported the onset of CAD at a median age of 55 years compared to 65 years in Western countries. Malaysians were reported to develop ACS at a mean age of 58.7 years compared to 63.4 to 68 years in most developed countries^3–5^. Nevertheless, the definition of “Premature Coronary Artery Disease” (PCAD) was not consistent across studies due to the fact that there is no universally accepted age threshold for the determination of PCAD.

The risk of CAD rises with age in both males and females, although the risk rises faster in females, males are significantly more likely to develop CAD. Age, smoking, hypertension (HTN), gender, hypercholesterolaemia (HC), and diabetes mellitus (DM) are the main risk factors for developing CVD^6,7^. Additionally, patients with CAD also commonly bearfamily history of first-degree relatives of myocardial infarction (MI)^8^, obesity and dyslipidaemia^9^. Factors such as smoking, HC and family history of CAD are more prevalent among PCAD patients in particular^10^.

World Health Organization (2018) reported that in 2016, 31% of global fatalities, which was estimated to be 17.9 million deaths were due to CVD, with heart attack and stroke representing 85% of those deaths. Most Asian countries besides Japan, South Korea, Singapore and Thailand, have higher age-adjusted mortality from CVD compared to Western countries^11^. The Department of Statistics Malaysia reported that out of 100,000 medically certified fatalities in 2020, ischaemic heart disease remained as the leading cause of death, accounting for 17.0% of the total death^12^. A local study reported that the proportion of PCAD (male: < 55 years; females: < 65 years) was 55% among patients undergoing coronary angiograms from 2002 until 2004^13^. A more recent report from the National Heart Association of Malaysia (NHAM) found the prevalence of PCAD (age < 50 years) to be 24.5%^3^. Many studies have reported the coronary risk factors (CRFs) of patients who had undergone percutaneous coronary intervention (PCI) in Malaysia^14–16^. However, the cut-off age or the definition for PCAD in many studies locally and globally differs from each other and may lead to multiple irresolute prevalence and risk factors outcomes. Besides, reports on the prevalence and risk factors of PCAD, depending on different age cut-offs for PCAD among PCI patients are limited. Hence, the first objective in this study is to report the proportion of PCAD according to different age cut-offs that define PCAD among patients who underwent PCI procedures. This study also aims to analyse the significant PCAD risk factors based on different age cut-off among patients who underwent PCI procedures.

## Methods

### Study design and population

This is a retrospective study including patients who underwent Percutaneous Coronary Intervention (PCI) procedures between 2007 and 2018 at National Heart Institute (NHI) and UiTM Specialist Clinics (Cardiology and Lipid Clinics). Data was collected according to the earliest available data in the PCI electronic database. Malaysian citizens (age ≥ 16 years old) with at least one of the following presentations of CAD were included:

1. Acute ST-segment– elevation myocardial infarction (STEMI),
2. Non–ST-segment–elevation myocardial infarction (NSTEMI),
3. Stable/unstable angina pectoris with angiographically proven coronary disease

Those with repeated entry between 2007 and 2018, below the age of 16 years old, with unavailable age records and citizenship were excluded from the study.

### Medical history and clinical variables

Hypercholesterolemia (HC), HTN, DM and other comorbidities were considered present based on documented information from the medical history in the database or if there is lipid/cholesterol lowering, antihypertensive or antidiabetic medications that were prescribed upon admission. Hypercholesterolemia (HC) was defined as those with history or current known HC with/without lipid/cholesterol lowering treatment. Hypertension (HTN) was defined as systolic/diastolic level of ≥ 140/90 mmHg. Diabetes mellitus (DM) was defined as those with history or current known DM with/without anti-diabetic medication.

Smoking status and family history of PCAD were obtained based on documented information in the electronic medical record, data were collected upon admission through consultation. The family history of PCAD was defined as patients with first -degree relatives who has known history or current CAD. Body-mass-index (BMI) status was calculated and recorded according to height and weight documented prior to the PCI procedure.

### Classification of CAD

The recruited patients were divided into premature CAD (PCAD) and mature CAD (MCAD) according to different age cut-off. These age cut-offs were chosen based on commonly reported age that defines PCAD according to literatures^17–20^. PCAD and MCAD were classified into age cut-offs as follows:

**Table:** 

Groups	PCAD	MCAD	References
A	Males/females: < 45 years	Males/females: ≥ 45 years	Aggarwal et al.^17^
B	Males: < 50 years; Females: < 55 years	Males: ≥ 50 years; Females: ≥ 55 years	Vikulova et al.^18^
C	Males: < 55 years; Females: < 60 years	Males: ≥ 55 years; Females: ≥ 60 years	Nordestgaard et al.^19^
D	Males: < 55 years; Females: < 65 years old	Males: ≥ 55 years; Females: ≥ 65 years old	Arnett et al.^20^

### Statistical analysis

Data were analysed using IBM SPSS Statistics version 26 (IBM, NY, USA). Continuous data were presented as means and standard deviation (SD) [for parametric test] or median and interquartile range (IQR) [for non-parametric tests], while categorical data were presented as percentages. The significance of differences between the numerical variables was determined by Independent T test (for parametric tests) or Mann–Whitney test (for non-parametric tests). The significance of association between categorical variables was determined by using Chi-squared test^21^. All final analyses with *p*-value < 0.05 were considered as statistically significant.

Logistic regression analysis was performed to determine the independent predictors of factors associated with PCAD for each age groups. All variables (CRFs) were subjected to simple logistic regression. Variables with *p*-value < 0.25 and clinically relevant were then included in multiple logistic regression analysis. Two-way interactions of each CRFs were checked, and relevant significant variables were included in the model. The independent variables were included into multiple logistic regression and multicollinearity were checked. Hosmer Lemeshow Goodness of Fit test^22^, the classification table and receiver operator characteristic curve was performed to test the fitness of model. All final analyses with *p*-value < 0.05 were considered as statistically significant.

Machine learning technique was performed to determine the influence factors of PCAD using decision-tree method (RapidMiner, Germany). The decision tree (DT) model is an algorithm derived from information theory. The classification rule in DT is created by repeatedly dividing the data into increasingly more homogeneous groups, with respect to the variable of interest, a method defined as recursive partitioning^23^. The values calculated using this method (weight attributes) was determined with respect to the feature importance for the given attribute, in which the higher the weight of an attribute, the more relevant it is considered. Prior to the analysis of DT, Synthetic Minority Oversampling Technique (SMOTE) application^24^ in the RapidMiner software was used to resolve imbalance data issue. The performance of DT model was evaluated according to the accuracy, sensitivity, specificity and precision values.

Conventional CRFs consisting of gender, ethnicity, smoking status, HC, HTN, DM, family history of PCAD, total cholesterol (TC), low-density lipoprotein (LDL-c) level, BMI status (overweight and obesity) and other comorbidities were included in the analysis of DT. In addition to the contribution factors (influence factors) of PCAD; the accuracy, precision, sensitivity and specificity of the model were also determined in the process.

### Ethics

The ethical approval was obtained from participating organisations through the respective Institutional Research Ethics Committees of Universiti Teknologi MARA (UiTM) [ref: 600-RMI (5/1/6)] and Institut Jantung Negara (IJN) [IJNEC/03/2012^6^] prior to commencement of the study. All patient data were derived from the PCI-database of Cardiology Clinic (UiTM) and IJN. Both Institutional Research Ethics Committees had approved data analysis and waived the informed consent. The Specialist Cardiology Clinic (UiTM) and Institut Jantung Negara (if this is the IRB or ethics committee) approved waived informed consent. All data were analysed anonymously. The study was conducted in accordance with the Declaration of Helsinki.

## Results

A total of 29,241 patients who had their first PCI from the year 2007 to 2018 were included in the study (Table 1). Majority of patients admitted were males (82.6%) and more than half were Malays (58.0%). Half of the cases were categorised as chronic stable angina upon admission (50.1%) and about 90% were referred for PCI as an elective procedure (Table 2).

**Table 1:** Demographics of the subjects of this study (n = 29,241).

Parameters	Frequency
Overall cases	29,241
Age (years)	58.2 ± 10.2
Age range (years)	
Males	16–90
Females	19–98
Overall age range (years)	16–98
Gender	
Males	24,161 (82.6)
Females	5080 (17.4)
Ethnicity	
Malay	16,961 (58.0)
Chinese	4346 (14.9)
Indian & others	7934 (27.1)

Values are presented as; n (percentage) and mean age ± standard deviation (SD) (years).

**Table 2:** Cardiac status of patients who underwent PCI (n = 29,241).

Parameters	Frequency
Angina type^a^	
Typical	56 (0.2)
Atypical	3420 (12.3)
Chronic stable angina	13,991 (50.1)
Unstable angina	3560 (12.8)
No angina	6891 (24.7)
PCI status	
Elective	26,122 (89.3)
NSTEMI/ UA	1219 (4.2)
STEMI	1900 (6.5)
NYHA^a^	
I	16,346 (58.2)
II	10,227 (36.4)
III	1247 (4.4)
IV	257 (0.9)
CCS^a^	
CCS 0	2493 (9.0)
CCS 1	10,152 (36.7)
CCS 2	12,752 (46.1)
CCS 3	1681 (6.1)
CCS 4	560 (2.0)
Killip class^a^	
I	5332 (18.4)
II	2922 (10.1)
III	153 (0.5)
IV	382 (1.3)
Not applicable/not available	20,182 (69.7)

Values are presented as; n (percentage).

*NSTEMI* non–ST-segment–elevation myocardial infarction, *UA* unstable angina, *STEMI* ST-elevation myocardial infarction, *NYHA* New York Heart Association, *CCS* Canadian Cardiovascular Score.

^a^Patients with no data was excluded from the analysis.

**Figure 1:**
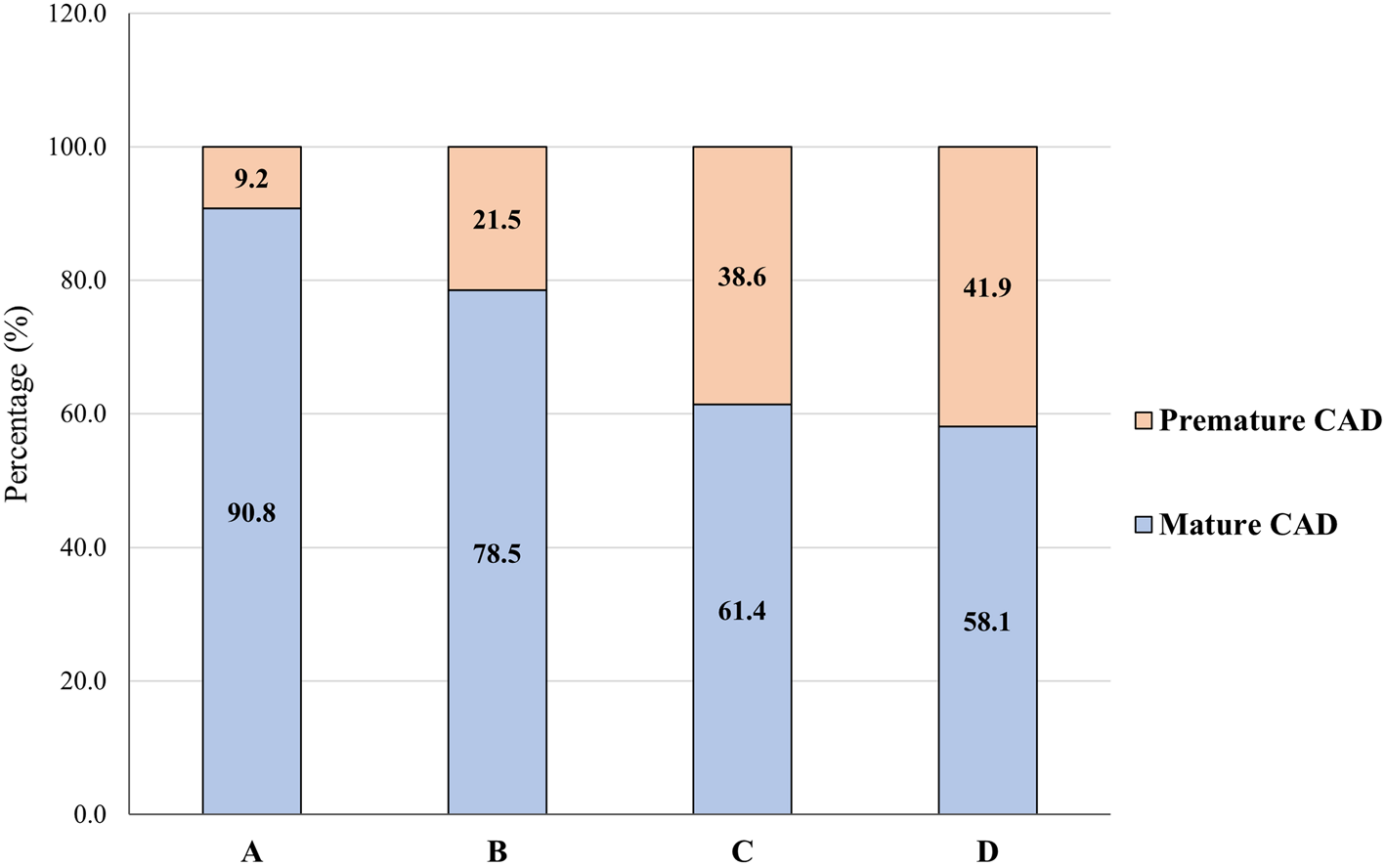
Proportions of premature CAD (PCAD) based on different age cut-offs: (**A**) < 45 years, (**B**) Males: < 50 years; Females: < 55 years, (**C**) Males: < 55 years; Females: < 60 years and (**D**) Males: < 55 years; Females: < 65 years old.

Figure 1 shows the proportion of PCAD depending on different age cut-offs. The youngest age cut-off (age group A) in this cohort shows that the proportion of PCAD was 9.2%, followed by 21.5%, 38.6% and 41.9% (age group B, C and D, respectively).

Table 3 shows the association between the risk factors and comorbidities with PCAD, comparing between PCAD and MCAD across each age group. The results showed that gender is significantly associated with PCAD in age cut-offs (A) and (D) only. On the other hand, gender was not significantly associated with PCAD in group (B) and (C) [p = 0.113 and p = 0.192; respectively] and the ratio of males to females between PCAD and MCAD groups was similar.

**Table 3:** Risk factors and comorbidities of premature CAD and mature CAD across all groups (n = 29,241; respectively).

Parameters	A			B			C			D		
	Premature CAD (< 45 male/ female)	Mature CAD (≥ 45 male/ female)	p value	Premature CAD (< 50 male; < 55 female)	Mature CAD (≥ 50 male; ≥ 55 female)	p value	Premature CAD (< 55 males; < 60 females)	Mature CAD (≥ 55 males; ≥ 60 females)	p value	Premature CAD (< 55 male; < 65 female)	Mature CAD (≥ 55 male; ≥ 65 female)	*p* value
Proportion	2686 (9.2)	26,555 (90.8)	-	6296 (21.5)	22,945 (78.5)	-	11,287 (38.6)	17,954 (61.4)	-	12,258 (41.9)	16,983 (58.1)	-
Age	39.4 ± 4.3	60.1 ± 8.5	< 0.001	44.4 ± 5.4	61.9 ± 7.6	< 0.001	48.18 ± 6.109	64.43 ± 6.595	< 0.001	49.28 ± 6.971	64.56 ± 6.748	< 0.001
Gender												
Males	2495 (92.9)	21,666 (81.6)	< 0.001	5160 (82.0)	19,001 (82.8)	0.113	9285 (82.3)	14,876 (82.9)	0.192	9285 (75.7)	14,876 (87.6)	< 0.001
Females	191 (7.1)	4889 (18.4)		1136 (18.0)	3944 (17.2)		2002 (17.7)	3078 (17.1)		2973 (24.3)	2107 (12.4)	
Smoking^a^												
Non-smoker	610 (24.9)	10,968 (47.9)	< 0.001	2020 (35.6)	9558 (48.6)	< 0.001	3821 (37.9)	7757 (50.9)	< 0.001	4648 (42.5)	6930 (48.2)	< 0.001
Current smoker	1196 (48.9)	5443 (23.8)		2244 (39.6)	4395 (22.4)		3639 (36.1)	3000 (19.7)		3657 (33.4)	2982 (20.7)	
Former smoker	642 (26.2)	6470 (28.3)		1406 (24.8)	5706 (29.0)		2628 (26.1)	4484 (29.4)		2643 (24.1)	4469 (31.1)	
Hypercholesterolaemia^a^												
Yes	1802 (67.8)	19,023 (72.0)	< 0.001	4365 (69.9)	16,460 (72.1)	0.001	7992 (71.3)	12,833 (71.9)	0.278	8704 (71.5)	12,121 (71.8)	0.587
No	857 (32.2)	7386 (28.0)		1879 (30.1)	6364 (27.9)		3220 (28.7)	5023 (28.1)		3474 (28.5)	4769 (28.2)	
Hypertension^a^												
Yes	1486 (55.7)	20,338 (76.8)	< 0.001	3960 (63.2)	17,864 (78.1)	< 0.001	7562 (67.3)	14,262 (79.7)	< 0.001	8416 (68.9)	13,408 (79.2)	< 0.001
No	1180 (44.3)	6142 (23.2)		2306 (36.8)	5016 (21.9)		3679 (32.7)	3643 (20.3)		3795 (31.1)	3527 (20.8)	
Diabetes mellitus^a^												
Yes	971 (36.4)	14,322 (54.1)	< 0.001	2792 (44.6)	12,501 (54.6)	< 0.001	5354 (47.6)	9939 (55.5)	< 0.001	6027 (49.3)	9266 (54.7)	< 0.001
No	1695 (63.6)	12,154 (45.9)		3475 (55.4)	10,374 (45.4)		5888 (52.4)	7961 (44.5)		6186 (50.7)	7663 (45.3)	
Family history of premature CAD^a^												
Yes	616 (25.2)	3922 (16.0)	< 0.001	1316 (22.8)	3222 (15.2)	< 0.001	2233 (21.5)	2305 (13.8)	< 0.001	2386 (21.2)	2152 (13.7)	< 0.001
No	1833 (74.8)	20,656 (84.0)		4452 (77.2)	18,037 (84.8)		8149 (78.5)	14,340 (86.2)		8890 (78.8)	13,599 (86.3)	
BMI^a^												
Underweight (BMI < 18.5 kg/m^2^)	20 (0.8)	295 (1.2)	< 0.001	42 (0.7)	273 (1.3)	< 0.001	82 (0.8)	233 (1.5)	< 0.001	90 (0.8)	225 (1.5)	< 0.001
Normal (BMI 18.5–29.9 kg/m^2^)	241 (10.0)	3814 (16.0)		587 (10.4)	3468 (16.9)		1103 (10.9)	2952 (18.4)		1263 (11.5)	2792 (18.4)	
Overweight (BMI 23–24.9 kg/m^2^)	349 (14.5)	4399 (18.5)		819 (14.5)	3929 (19.1)		1604 (15.8)	3144 (19.6)		1743 (15.8)	3005 (19.8)	
Obese I (BMI 25–29.9 kg/m^2^)	991 (41.3)	10,345 (43.4)		2504 (44.3)	8832 (42.9)		4548 (44.8)	6788 (42.3)		4883 (44.3)	6453 (42.5)	
Obese II (BMI ≥ 30 kg/m^2^)	798 (33.3)	4967 (20.9)		1698 (30.1)	4067 (19.8)		2818 (27.7)	2947 (18.3)		3044 (27.6)	2721 (17.9)	
Systolic blood pressure (On medication)^a^	124.1 ± 20.8	135.8 ± 24.8	< 0.001	128.5 ± 23.3	136.5 ± 24.8	< 0.001	130.5 ± 24.1	137.5 ± 24.8	< 0.001	131.6 ± 24.5	137.1 ± 24.6	< 0.001
Diastolic blood pressure (On medication)^a^	78.7 ± 14.3	76.3 ± 16.4	< 0.001	79.1 ± 17.5	75.8 ± 15.8	< 0.001	78.6 ± 15.8	75.2 ± 16.4	< 0.001	78.32 ± 16.9	75.16 ± 15.6	< 0.001
TC [mmol/L] (On medication)^a^	4.5 (3.7–5.5)	4.20 (3.5–4.9)	< 0.001	4.4 (3.7–5.3)	4.1 (3.5–4.9)	< 0.001	4.4 (3.7–5.2)	4.1 (3.5 -4.8)	< 0.001	4.4 (3.7–5.2)	4.1 (3.5–4.8)	< 0.001
LDL-c [mmol/L] (On medication)^a^	2.6 (2.0–3.4)	2.3 (1.8–2.9)	< 0.001	2.5 (1.9–3.3)	2.3 (1.7–2.9)	< 0.001	2.5 (1.9–3.2)	2.2 (1.7–2.9)	< 0.001	2.4 (1.9–3.2)	2.2 (1.7–2.8)	< 0.001
History of myocardial infarction^a^												
Yes	1605 (60.3)	12,465 (47.3)	< 0.001	3479 (55.6)	10,591 (46.5)	< 0.001	6002 (53.6)	8068 (45.3)	< 0.001	6387 (52.5)	7683 (45.6)	< 0.001
No	1058 (39.7)	13,885 (52.7)		2774 (44.4)	12,169 (53.5)		5202 (46.4)	9741 (54.7)		5782 (47.5)	9161 (54.4)	
Cerebrovascular disease^a^												
Yes	47 (1.8)	697 (2.6)	0.006	110 (1.8)	634 (2.8)	< 0.001	229 (2.0)	515 (2.9)	< 0.001	251 (2.1)	493 (2.9)	< 0.001
No	2631 (98.2)	25,807 (97.4)		6172 (98.2)	22,266 (97.2)		11,031 (98.0)	17,407 (97.1)		11,980 (97.9)	16,458 (97.1)	
Peripheral vascular disease^a^												
Yes	8 (0.3)	244 (0.9)	0.001	25 (0.4)	227 (1.0)	< 0.001	54 (0.5)	198 (1.1)	< 0.001	65 (0.5)	187 (1.1)	< 0.001
No	2670 (99.7)	26,260 (99.1)		6257 (99.6)	22,673 (99.0)		11,207 (99.5)	17,723 (98.9)		12,167 (99.5)	16,763 (98.9)	
Chronic kidney disease^a^												
Yes	66 (2.5)	1901 (7.2)	< 0.001	236 (3.8)	1731 (7.6)	< 0.001	537 (4.8)	1430 (8.0)	< 0.001	627 (5.1)	1340 (7.9)	< 0.001
No	2613 (97.5)	24,620 (92.8)		6047 (96.2)	21,186 (92.4)		10,729 (95.2)	16,504 (92.0)		11,610 (94.9)	15,623 (92.1)	

Values are presented as mean ± SD, or median and interquartile range (IQR) or n (percentage).

*CAD* coronary artery disease, *BMI* body-mass index, *TC* total cholesterol, *LDL-c* low-density lipoprotein cholesterol.

*Mann Whitney test.

^a^Patients with no data was excluded from the analysis.

Besides, there are a significant association between HC and PCAD in age groups (A) and (B) [p < 0.001]. However, the association was not significant in age groups (C) and (D) [p = 0.278 and p = 0.587; respectively]. Generally, there are significantly more HC cases among the MCAD in the younger age groups (A and B) compared to PCAD. Nevertheless, PCAD cases have significantly higher levels of TC and LDL-c compared to MCAD (p < 0.001).

In all age groups, smoking, HTN, DM, and family history of PCAD were significantly associated with PCAD. Evidently, in the younger age cut-off groups (A) and (B), current smoker was significantly the highest compared to non and former smoker among PCAD. In contrast, there is more non-smoker compared to current and former smoker among PCAD in the older age cut-off groups (C) and (D). In general, there are more current smoker among PCAD cases compared to MCAD in all age groups (p < 0.001).

There were significantly more hypertensive cases among MCAD compared to PCAD (p < 0.001) in all groups. Similarly, there were also more DM cases among MCAD compared to PCAD (p < 0.001). The pattern in all groups was similar in the sense that there were more non-DM cases compared to DM cases among PCAD, which shows that DM may be exclusive to older ages.

Besides, family history of PCAD was significantly higher among PCAD cases compared to MCAD in all age groups. There were more obese (BMI > 25 kg/m^2^) cases among PCAD compared to MCAD, but there were more MCAD cases who were overweight compared to PCAD (p < 0.001). In addition, MCAD cases carry significantly more cases of other comorbidities such as cerebrovascular disease, peripheral vascular disease (PVD) and chronic kidney disease (CKD) compared to those of PCAD (p < 0.001; respectively).

For the youngest age group (A), all conventional risk factors were significantly associated with PCAD. For age group (B), only gender was not associated with PCAD. There are two risk factors (gender and HC) that are not associated with PCAD for age group (C). On the other hand, only HC was not associated with PCAD in the oldest age cut-off (D). However, considering the cholesterol level was significantly higher among PCAD compared to MCAD in all age groups; only gender was shown to have no significance difference for the middle age groups (B and C).

**Figure 2:**
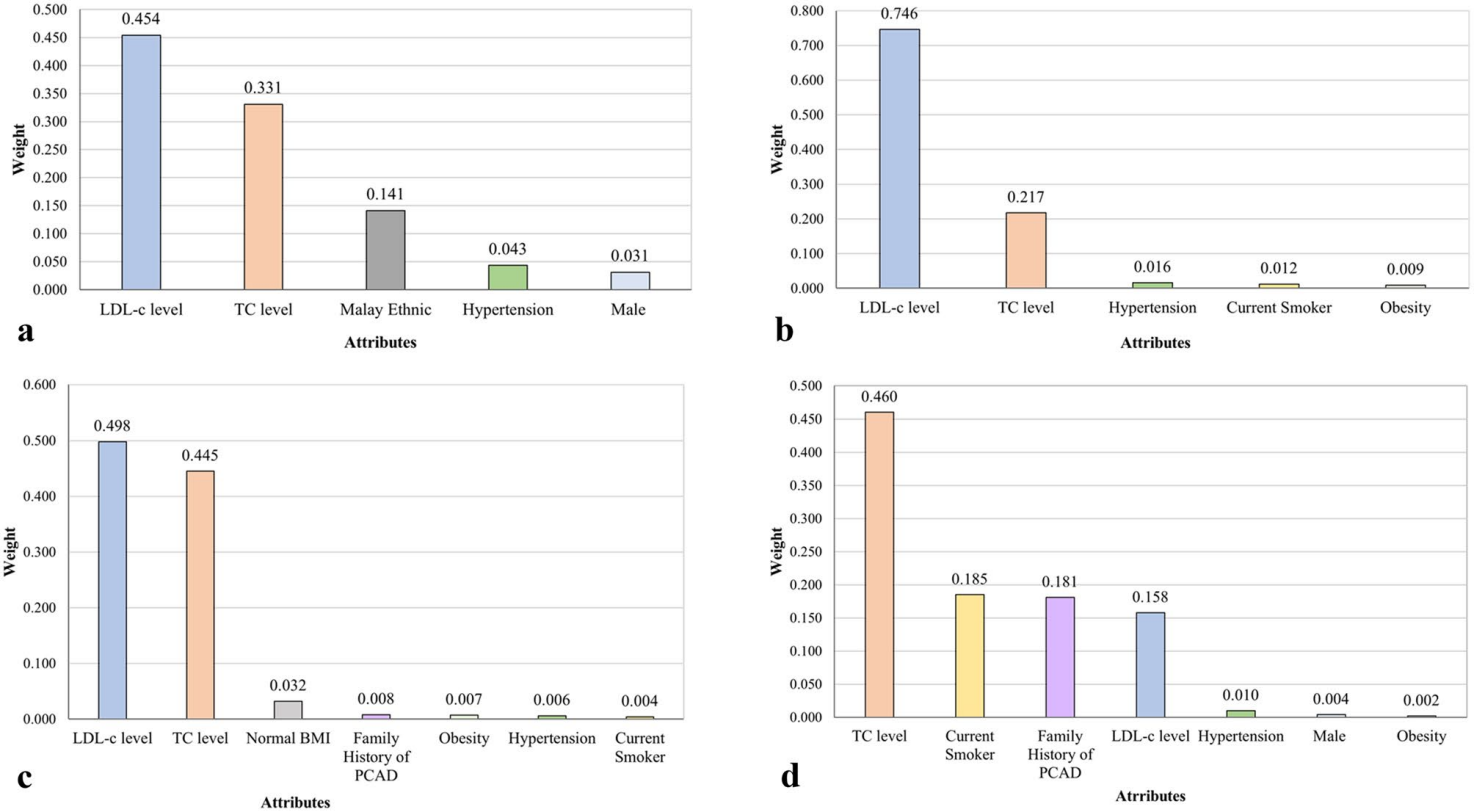
Ranking of risk factors of premature coronary artery disease across four age groups. (**a**) Age group A; Males/Females < 45 years, (**b**) Age group B; Males: < 50 years; Females: < 55 years, (**c**) Age group C; Males: < 55 years; Females: < 60 years and (**d**) Age group D; Males: < 55 years; Females: < 65 years old.

Using DT method, five attributes (CRFs) were selected for age groups (A) and (B), and seven attributes for age groups (C) and (D) (Fig. 2). These attributes were selected based on the risk factors that influenced PCAD the most, in ranking order. The diagnostic performance of DT method shows an average accuracy of 61.1%, sensitivity of 51.2%, specificity of 70.9% and precision of 63.9% (Table 4). Based on the accuracy and sensitivity, age group (D) has the highest accuracy (62.6%) and sensitivity values (57.2%). On the other hand, age group (A) has the highest values for specificity (77.9%) and precision (66.2%) compared to other age groups.

**Table 4:** The diagnostic performance of decision-tree method.

Age Groups	Accuracy (%)	Sensitivity (%)	Specificity (%)	Precision (%)	Classification error (%)	Attributes
A	60.56	43.20	77.92	66.17	39.44	5
B	60.97	55.84	66.11	62.23	39.03	5
C	60.25	48.63	71.87	63.35	39.75	7
D	62.64	57.21	68.07	64.18	37.36	7
Average	61.11	51.22	70.99	63.98	38.90	6

From the CRFs selected, it was apparent that LDL-c and TC levels were significantly important among PCAD across all age groups (Fig. 2). LDL-c level ranking first for groups (A), (B), (C) and fourth for (D), and TC level ranking second for (A), (B), (C) and first for (D). Besides, HTN was one of the CRFs that influence PCAD the most; selected across all age groups. Current smoking and obesity were also among the top; selected in three out of the four age groups.

However, despite LDL-c level ranking high in influencing PCAD, multiple logistic regression assessment eliminates LDL-c and positive HTN as the predictors of PCAD (Table 5). The analysis shows that out of all variables, only Malay ethnicity, current/former smoker, obesity, history of MI, positive family history of PCAD and elevated TC level were positively associated with PCAD that consistently appears across all age groups (p < 0.05), thus shown to be the independent predictors of PCAD. On the other hand, although HTN, DM and CKD also significantly associated with PCAD across all age group, the association was negative.

**Table 5:** Independent predictor of factors associated with premature coronary artery disease.

Parameters	A		B		C		D	
	Adjusted OR^a^ (95%CI)	p-value*	Adjusted OR^a^ (95%CI)	p-value*	Adjusted OR^a^ (95%CI)	p-value*	Adjusted OR^a^ (95%CI)	p-value*
Male	1.522 (1.232, 1.881)	< 0.001	NS	NS	NS	NS	NS	NS
Female	NS	NS	1.832 (1.635, 2.053)	< 0.001	1.764 (1.605, 1.939)	< 0.001	3.994 (3.630, 4.395)	< 0.001
Malay	1.129 (1.010, 1.263)	0.033	1.149 (1.063, 1.241)	< 0.001	1.160 (1.087, 1.239)	< 0.001	1.156 (1.083, 1.234)	< 0.001
Current/former smoker	1.799 (1.579, 2.049)	< 0.001	1.697 (1.550, 1.858)	< 0.001	1.722 (1.599, 1.853)	< 0.001	1.731 (1.607, 1.863)	< 0.001
Obesity	1.799 (1.597, 2.026)	< 0.001	1.899 (1.749, 2.063)	< 0.001	1.838 (1.717, 1.966)	< 0.001	1.835 (1.715, 1.963)	< 0.001
Hypertension	0.493 (0.441, 0.550)	< 0.001	0.524 (0.483, 0.569)	< 0.001	0.565 (0.525, 0.608)	< 0.001	0.571 (0.530, 0.615)	< 0.001
Diabetes mellitus	0.655 (0.586, 0.732)	< 0.001	0.810 (0.750, 0.875)	< 0.001	0.861 (0.807, 0.919)	< 0.001	0.871 (0.816, 0.930)	< 0.001
Personal history of MI	1.627 (1.459, 1.814)	< 0.001	1.431 (1.327, 1.542)	< 0.001	1.366 (1.283, 1.456)	< 0.001	1.368 (1.283, 1.457)	< 0.001
Cerebrovascular disease	NS	NS	0.604 (0.451, 0.808)	0.001	0.683 (0.547, 0.853)	0.001	0.673 (0.540, 0.838)	< 0.001
Peripheral vascular disease	NS	NS	NS	NS	0.596 (0.392, 0.909)	0.016	0.580 (0.384, 0.877)	0.010
Chronic kidney disease	0.494 (0.349, 0.698)	< 0.001	0.622 (0.514, 0.752)	< 0.001	0.803 (0.698, 0.924)	0.002	0.809 (0.704, 0.929)	0.003
Family history of PCAD	1.637 (1.453, 1.845)	< 0.001	1.513 (1.385, 1.652)	< 0.001	1.581 (1.461, 1.710)	< 0.001	1.607 (1.484, 1.739)	< 0.001
Total cholesterol	1.236 (1.188, 1.286)	< 0.001	1.221 (1.184, 1.258)	< 0.001	1.209 (1.176, 1.243)	< 0.001	1.224 (1.190, 1.258)	< 0.001
	Hosmer and Lemeshow goodness-of-fit test, p-value: 0.553; Percentage prediction: 90.7%		Hosmer and Lemeshow goodness-of-fit test, p-value: 0.164; Percentage prediction: 78.1%		Hosmer and Lemeshow goodness-of-fit test, p-value: 0.825; Percentage prediction: 64.4%		Hosmer and Lemeshow goodness-of-fit test, p-value: 0.104; Percentage prediction: 64.7%	

A: < 45 years, B: Males: < 50 years; Females: < 55 years, C: Males: < 55 years; Females: < 60 years and D: Males: < 55 years; Females: < 65 years old.

*OR* odds ratio, *CI* confidence interval, *PCAD* premature coronary artery disease.

*Statistically significant at ɑ = 0.05. Model is fit, model assumptions are met and no multicollinearity problems.

^a^Statistical test: Multiple Logistic Regression.

## Discussion

To understand coronary risk factors, the key element to reach the goals of management, treatment and prevention of CAD is by identifying the early point at which these actions must take place. It is worth noting that the distinction between early and late CAD can vary depending on the location and specific study being conducted. Previously, Abderrahman, Al-Abdallat^25^ reported a definition of the age-threshold for PCAD and mature CAD among males by using the age-related differences in the prevalence of coronary thrombosis and the presence of myocardial fibrosis, as reported via autopsy. The study emphasised thrombosis as the main underlying mechanism of death in PCAD. They deduced that PCAD constituted the cases suffering from the heart attack or died due to cardiac attack at the age < 49 years, and the mature disease is the one affecting people at the age > 54 years old. Nevertheless, the specific definition of premature CAD is difficult to be determined as the risk factors are variables between different populations and age groups. To the best of our knowledge, this study is the first to identify and compare the frequency and risk profiles based on different age cut-offs.

In this present study, we identified the proportions of PCAD based on several commonly used age threshold that defines PCAD based on literatures^17–20^. The average proportion of PCAD was 28%, ranging between 9 and 42%. Even though other study reported similar prevalence (9.2% vs 11.6%) using the similar age cut-off (A)^15^, or even much higher prevalence (24.5%; age < 50 years)^3^; some studies reported a lower prevalence compared to the current study. For instance, for age cut-off (A), Aggarwal and colleagues^17^ reported that 1.2% of CAD cases were PCAD. For age cut-off (B), other study reported a lower frequency (9.4%), compared to ours (22%)^18^. For age cut-off (D), a German study reported a lower prevalence of PCAD (37.2%) compared to the current study (41.9%)^26^. A previous local study confirms that among those who underwent PCI, Malaysians were substantially younger and had a greater incidence of risk factors compared to those in other countries' PCI databases^27^. Accordingly, studies using the younger age cut-off reported a lower prevalence of PCAD and the proportion increases as the age cut-off increases. In short, the proportion may vary depending on the age cut-off chosen.

This could possibly be explained by the fact that Malaysia is a culturally diversified country that consumes combinations of unhealthy diets of traditional dishes of multiple ethnicities over the years. This leads to the development of multiple atherosclerotic diseases and other comorbidities that eventually leads to the development of early onset CAD within this population. This factor may demonstrate how social, and environmental variables could influence the development of cardiovascular diseases.

Smoking is an established risk factor associated with PCAD and CAD^28,29^. In this present study, smoking is more prevalent among PCAD compared to MCAD. Regression and DT analysis suggests a strong association between smoking and PCAD, which is in concordance with the available literatures^30,31^. Regression analysis has demonstrated that the predictors (risk factors) are independent of each other. In contrast, machine learning; specifically DT analysis shows the factors that influence PCAD the most, in ranking order, where the risk factors were dependent of each other. Nevertheless, the results found that both methods strongly suggest a significant association between smoking and PCAD.

Besides smoking, family history of PCAD was also evidently more prevalent among PCAD compared to MCAD, which is similar to other studies^30,32^, However, the DT analysis only selected smoking in two out of the four age groups. (C and D). Nevertheless, positive family history of PCAD is one of the independent predictors of PCAD across all age groups. The result is supported by other findings that report family history of PCAD as an important predictor of PCAD^33^ and CHD in general^34^. A study reported the odds ratios (ORs) for several diseases including CVD, which exceed 2 for people with one affected family member (first degree) and exceed 4 for a lot of the diseases if there is > 1 affected family members^35^. Despite the awareness of the importance of genetic and parental history on the development of CVD, both are still not a routine practice in the determination of CVD risks^36^.

Aside from not being a routine practice, there is also a lack of in-depth dive into the actual history and diseases underlying among family members in normal clinical setting. In a recent study examining family health history within routine primary care consultations, it was discovered that the majority of family history conversations that took place during these consultations lasted less than three minutes. The discussions, which centered around either non-specific inquiries or specific conditions, were found to be typically brief in nature. These results indicate that there may be room for improvement in terms of the depth and scope of family health history conversations within primary care settings. Moreover, a significant number of patients responded with an uncooperative answer or simply replied ‘no,’ which posed a challenge in obtaining a comprehensive and accurate family history. This was often attributed to the personal preference of patients where they preferred not to disclose such information^37^. On the other hand, a detailed family history of three generations of relatives, health issues with age of onset, the age and cause of death for each family member was estimated to lasts up to 30 min^38^. Besides, identifying parental history based on “yes” or “no” was deemed an oversimplification that fails to determine the pivotal details that affects the risk such as the relatives’ age of onset of disease, relationships with the patients, and actual number of relatives affected with the disease^36,39–42^. Furthermore, the importance of detailed family history was proven by the fact that siblings of those with CVD have about 40% risk increase, while offspring of parents with premature CVD have a 60% (maternal history) to 75% (paternal history) risk increase^43^. This further shows the importance of a thorough examination by trained or experienced general practitioners.

The findings in the present study shows that HTN is significantly more prevalent among MCAD, and regression analysis shows a negative association with PCAD, where individuals with no-HTN have an average of 2.00 times the odds to have PCAD compared to those with positive HTN (across all groups). Interestingly, DT analysis selected HTN in all age groups as the top factors that influence PCAD besides LDL-c and TC levels. Hypertension is a well-established risk factor for cardiovascular events and several clinical trials have also found a robust, continuous, and linear relationship between high blood pressure (BP) and CVD^44–47^. Lowering elevated BP has shown to reduce coronary risk^48^. Thus, it is best to assume that HTN in general is almost always comes in pair with CAD, irrespective of the age and gender. In fact, World Health Organization (WHO) reported that besides significantly increases the risks of heart, brain, kidney and other diseases, approximately 1.3 billion adults aged 30–79 years worldwide have HTN and only 21% of known cases have it under control^49^.

The regression analysis also shows that besides HTN; DM and CKD also are negatively associated with PCAD in all age groups. Despite many studies and guidelines reporting these factors as strong risks of developing CAD and PCAD; and even proposed as acceleration mechanisms of CAD^10,50–54^; HTN, DM and CKD were significantly more prevalent in MCAD compared to PCAD patients. The results might be explained by simply the increasing in age, since these factors are more commonly developed at much older age, as the number of risk factors and their severity increase with age^13,15^.

A previous study found that HTN and diabetes mellitus (DM) to be associated with older CAD onset, which might be explained by the fact that those diagnosed with these diseases might have received treatment earlier in life^55^. Other studies showed that DM and HTN were associated with a lower risk of CAD in young patients compared with elderly patients as the occurrence of DM and HTN were more frequent in those with late-onset CAD compared to young CAD^26,56–62^.

Hypertension is highly associated with increasing age; hence the incidence is more common in older people^63^. The main factor is due to the complex mechanism which involves slow grade processes that take years to develop^62,64^. The arterial vasculature changes structurally and functionally due to aging. The elastic lamellae of the arteries break down with time, and the aorta develops intimal hyperplasia. The reduced capacitance and limited rebound of the stiffened arteries makes it challenging to accept variations in volume during the cardiac cycle. Both systolic blood pressure (SBP) and diastolic blood pressure (DBP) rise with age, but after the age of 60, central arterial stiffness predominates; hence, SBP keeps rising while DBP starts to fall. As a result, the pulse pressure widens and there is isolated systolic HTN. The widened pulse pressure rises with age regardless of mean blood pressure or any other determining factors^65,66^. Many other underlying mechanisms of HTN such as the neurohormonal dysregulation, mechanical hemodynamic changes, autonomic dysregulation, as well as the aging kidney leads to the common occurrence of HTN in older adults^67^.

Similarly, age is also an important risk factor for DM and CKD and almost 50% of individuals with DM are at age ≥ 65 years^68^. Alterations in both insulin sensitivity and insulin secretion in elderly adults eventually impair glucose tolerance, which results in DM^69^. Chronic systemic inflammation, oxidative stress, DNA damage, decreased mitochondrial function, tissue dysfunction and cellular senescence are all factors that are exacerbated by aging and contribute to the development of metabolic diseases^70^, which may further explains our findings. All in all, these results might be explained by simply the increasing age, since these factors are more commonly developed at older age, as the number of risk factors and their severity increase with age^13,15^.

Even though males were more prevalent, all the analysis shows a weak association between gender (especially males) and PCAD in this study, though males are still prone to have CAD compared to females, as shown in DT analysis. However, regression analysis shows that females were an independent predictor of PCAD among the older age cut-offs (age groups B, C and D). The potential explanation for this is that post-menopause for females is one of the risk factors of CAD^71,72^ It could also be explain by the fact that postmenopausal females have higher plasma TC, LDL-c, very low-density lipoprotein cholesterol (VLDL-c), and TG levels^73,74^ which may lead to CAD. Besides, even though CAD develop much later in most cases for females, CAD incidence and mortality in males was higher compared to females^75^. In addition, Mumford et al. reported that the prevalence of CVD among females aged 20–39 years is half of that for males within the same age group (females: 7.8%; males: 15.9%) and the gender disparity in CVD narrows with increasing age as the prevalence among women increases^76^ which can be due to menopause.

With a relatively good diagnostic performance, the DT analysis selected LDL-c, TC and HTN as the topmost important factors that contributes to PCAD. This is expected since LDL-c was the essential of hypercholesterolaemia (including non-HDL cholesterol) and proven to be the cause of the formation of fatty and fibrous lesions in the arterial walls that leads to atherosclerosis^77,78^. Total cholesterol level also was shown to be highly associated with CVD risk among young adults^79^ and dyslipidaemia was reported to be among the most common modifiable risk factors in over one million young adults with MI in the United States^80^. Furthermore, a comprehensive analysis of a nationwide epidemiological database, comprising of over one million young adults from Japan demonstrated a close relationship between lipid profiles and subsequent CVD^81^.

The present study also showed how the DT analysis rank the risk factor in order of utmost influenced to PCAD. Interestingly, elevated LDL-c level rank the first for the first three younger age groups (A, B and C), but ranked fourth for the oldest age group (D). The possible explanation for this could be that a lot of these patients have been treated with lipid lowering therapy, probably for a long duration prior to the PCI procedure. Studies also have proven that lipid lowering therapy is effective in lowering LDL-c level in older adults^82^. Hence, shows that LDL-c can decrease with age^83^.

However, for the determination of independent predictors of PCAD in this study, LDL-c variable was eliminated upon multiple regression analysis—consistently across all age groups, while TC remains in the analysis. The level of LDL-c is often measured using calculation; most commonly using Friedewald equation, instead of direct measurement which may give inaccurate results^84,85^. Besides, even direct LDL-c assay is dependent on the proprietary chemical-based methods instead of ultracentrifugation. Another possible explanation is that the concentration of LDL-c only presents the amount of cholesterol by-product in LDL particles without other lipoprotein fractions such as Lp(a) or VLDL; which play a big role leading to atherosclerosis^86^. Those factors might explain why LDL-c level alone may not reliable to predict CAD. Nevertheless, national and international guidelines have always focussed on targeting LDL-c level and statin therapy has been widely promoted as the primary approach of treatment and the management of CAD/CVD^51,53,87^.

### Limitations

We acknowledge several limitations in this study. We have not implemented any genetic testing on genes that are commonly associated with CAD^88^ and familial hypercholesterolaemia (FH). Those with FH have lifelong exposure to HC, hence high LDL-c level since childhood and this elevated LDL-c leads to atherosclerosis which in turn increases the risk of developing PCAD. In fact, FH is long known to be a predisposing cause of PCAD^89,90^. We have also not measured the level of Lipoprotein(a) [Lp(a)] and serum gamma-glutamyl transferase (GGT); which was reported to be able to predict CAD in young patients^91^. An established and well-known gene that was reported to be an independent genetic risk marker for atherosclerosis and CVD is *LPA* that encodes apolipoprotein (a) and gives rise to Lipoprotein(a) particles. A previous genetic study has been instrumental in establishing Lp(a) as a significant risk biomarker for CAD^92,93^.

Next, we were unable to obtain the data for high-density lipoprotein cholesterol (HDL-C), triglyceride (TG), blood glucose, HbA1c levels as well as a complete drug history to support our results due to missing information and insufficient data collection at data entry level. Besides, the data reported in this study was collected upon admission for the PCI procedure. Thus, there is lack of information on the duration of certain risk factors such as DM and HTN.

There are several factors contributing to the fact that there are more patients with CAD who did not receive PCI. Such factors include financial difficulties, diagnosed as mild or moderate disease, choosing another form of medical treatment, or went for Coronary Artery Bypass Graft (CABG) instead. Besides, there might be potential information bias due to missing data that may introduce bias in the performance of prediction model. However, we have excluded the missing data from the chi-squared analysis, declaring the data as missing during logistic regressions, as well as implementing Synthetic Minority Oversampling Technique (SMOTE) application to solve data imbalance for DT method.

Future study with better approached in terms of data collection and information confirmation is also highly recommended. Also, a future study with a larger cohort of the general population, or those with specific types of CAD such as obstructive CAD, nonobstructive CAD or spontaneous coronary artery dissection and those underwent other procedure such as CABG is greatly recommended to identify the prevalence of PCAD or CAD.

## Conclusion

The proportion of PCAD among PCI-treated patients is high, ranging from about 9–40%, depending on age cut-offs used. We found that irrespective of the age cut-off for the classification of PCAD, LDL-c, TC and HTN are the most significant CRFs in PCAD across all age categories. Despite some remarkable differences in clinical characteristics between PCAD and MCAD, the four age cut-off groups did not differ significantly in the majority of the clinical outcomes and shares similar profiles of independent predictors. Future studies are warranted to investigate the potential genetic and other possible factors involved in the association between PCAD and coronary risk factors. Early prevention, detection and management of modifiable risk factors are highly warranted to prevent CAD among young individuals.

## Data Availability

The datasets generated and/or analysed during the current study are not publicly available due to patients’ clinical data being confidential and sensitive personal health information, which are protected by Malaysia Medical Act 1971. However, de-identified data are available from the corresponding author on reasonable request.
